# IMPT versus VMAT for Pelvic Nodal Irradiation in Prostate Cancer: A Dosimetric Comparison

**DOI:** 10.14338/IJPT-18-00048.1

**Published:** 2019-03-21

**Authors:** Thomas J. Whitaker, David M. Routman, Heather Schultz, William S. Harmsen, Kimberly S. Corbin, William W. Wong, Richard Choo

**Affiliations:** 1 Department of Radiation Oncology, Mayo Clinic, Rochester, MN, USA; 2 Department of Radiation Oncology, Mayo Clinic, Scottsdale, AZ, USA

**Keywords:** proton therapy, IMRT, VMAT, dosimetry, prostate cancer

## Abstract

**Purpose:**

To compare dosimetric data of the organs at risk (OARs) and clinical target volumes (CTVs) between intensity-modulated proton therapy (IMPT) and volumetric-modulated arc therapy (VMAT) for patients undergoing prostate and elective, pelvic lymph node radiotherapy in the setting of unfavorable, intermediate and high-risk prostate carcinoma.

**Methods and Materials:**

A study of moderately hypofractionated proton therapy (6750 centigray [cGy] in 25 fractions) is in progress for unfavorable, intermediate and high-risk prostate cancer where treatment includes an elective pelvic nodal CTV (4500 cGy in 25 fractions). Ten consecutively accrued patients were the subjects for dose-volume histogram comparison between IMPT and VMAT. Two treatment plans (IMPT and VMAT) were prepared for each patient with predefined planning objectives for target volumes and OARs. The IMPT plans were prepared with 2 lateral beams and VMAT plans with 2 arcs.

**Results:**

The CTV coverage was adequate for both plans with 99% of CTVs receiving ≥ 100% of the prescription doses. Mean doses to the bladder, rectum, large bowel, and small bowel were lower with IMPT versus VMAT. Mean femoral head dose was greater with IMPT. The percentage of volumes of rectum receiving ≤ 47.5 Gy, large bowel receiving ≤ 27.5 Gy, small bowel receiving ≤ 30 Gy, and bladder receiving ≤ 37.5 Gy was less with IMPT versus VMAT, largely because of reduction in the low-dose “bath” associated with VMAT.

**Conclusions:**

In the setting of prostate and elective, pelvic nodal radiotherapy for prostate cancer, IMPT can significantly reduce the dose to OARs, in comparison to VMAT, and provide adequate target coverage.

## Introduction

External beam radiotherapy (RT) has been widely used for the treatment of high-risk, clinically localized prostate carcinoma. Clinical target volumes of RT for high-risk prostate cancer often include the regional pelvic lymph nodes, because of the increased risk of nodal metastasis, along with the prostate and seminal vesicles. In addition, most large, published, randomized trials evaluating the efficacy of RT, with or without androgen-deprivation therapy, approached RT in this manner [1–6].

RT techniques have evolved rapidly with the introduction of intensity-modulated RT (IMRT), including more-recent techniques, such as volumetric modulated arc therapy (VMAT), an arc-based approach to IMRT. Although IMRT provides a more-conformal dose distribution to target volumes in comparison to 3 Dimensional conformal RT, IMRT spreads out the integral dose over a large volume of healthy tissue with significant dose to organs at risk (OARs). Proton therapy may provide an advantage over IMRT in sparing nontarget, healthy tissue while maintaining target coverage, given its characteristic dose distribution and Bragg peak with decreased exit dose. This dosimetric gain of proton therapy may be further amplified when clinical target volumes expand to a larger area increasing the volume of nearby OARs. Such an example is pelvic nodal irradiation for high-risk prostate carcinoma.

More recently, intensity modulated proton therapy (IMPT) has been incorporated into clinical practice, in which discrete spot scanning with a proton pencil beam delivers the dose from a highly conformal treatment plan. However, few studies have investigated dosimetric differences between IMPT and VMAT in the setting of elective pelvic nodal coverage for patients undergoing RT for prostate cancer. We aimed to perform such a comparison in a recent cohort of patients treated on a prospective study, NCT02874014 [7]. Specifically, we provide a dosimetric comparison between IMPT and VMAT for target coverage and dose to OARs for patients undergoing definitive prostate RT, which included elective pelvic nodal irradiation.

## Methods

A study of moderately hypofractionated proton therapy for patients with unfavorable, intermediate or high-risk prostate cancer completed accrual between August 2016 and November 2018. The study was approved by our institutional review board and was registered at clinicaltrials.gov (NCT02874014). Staging work-up included a bone scan and computed tomography (CT) or magnetic resonance imaging (MRI) scan of the abdomen and pelvis. Patients with clinical evidence of pelvic nodal or distant metastasis were ineligible for the study. In this single-arm, prospective study, the clinical target volumes (CTVs) included the regional pelvic lymph nodes (CTV low), as well as the prostate and the seminal vesicles (CTV high). The CTV high and CTV low received 6750 centigray (cGy) radiobiologic equivalent (RBE) and 4500 cGy (RBE), respectively. The two dose levels were delivered simultaneously in 25 fractions over 5 weeks. The first 10 enrolled patients provide the basis for this dosimetric study.

As part of the standard of care for unfavorable, intermediate or high-risk prostate cancer, patients also received adjuvant androgen deprivation therapy for 6 to 36 months. Androgen deprivation therapy started 2 months before the beginning of RT and consisted of bicalutamide 50 mg by mouth once daily for 2 to 4 weeks and a luteinizing hormone-releasing factor agonist (goserelin or leuprolide).

### Simulation

Four carbon fiducial markers were implanted into the prostate gland. These implanted carbon markers were used for daily image guidance. Patients were simulated with a full bladder. A planning CT scan was performed with the patient in supine position. An indexed knee cushion and a custom vacuum-lock bag were used to immobilize the legs and feet. The leg immobilization reduced daily setup variation, particularly variation in foot rotation. A scout CT scan was obtained to adjust rotation and bony alignment of the patient. The patient was then scanned from above the iliac crests to the mid femur with 2-mm slice thickness. Shortly after CT simulation was completed, MRI of the pelvis (MR simulation) was obtained with the same setup described above.

### Volume Definition

Both CT and MRI images were then imported into the Eclipse treatment planning system (Varian Medical Systems, Palo Alto, California), and the 2 data sets were fused, with the implanted, intraprostatic carbon markers as references.

The CTVs were delineated by the attending radiation oncologist, with MRI images as a supplemental tool for delineating the CTV high. The CTVs were defined according to the consensus guidelines and current protocols of the Radiation Therapy Oncology Group (RTOG) [8]. The CTV high consisted of the prostate and the seminal vesicles. The extent of seminal vesicles to be included in CTV high was determined at the discretion of the attending physician and was based on clinicopathologic features of the malignancy. The CTV low consisted of the regional pelvic lymph nodes and included a 7-mm margin in 3-dimensions to the iliac vessels and a 10-mm margin anteriorly from the anterior sacral bone for presacral nodes. Adjacent healthy organs (such as the rectum, small bowel, large bowel, and bladder), pelvic musculature, and bones were carved out from the CTV low. The CTV low included the obturator, external iliac, and proximal internal iliac and distal common iliac nodes up to a level corresponding to the sacral promontory or L5 to S1 junction. The presacral nodes from the sacral promontory to S3 were included, depending on whether the dose constraints to the rectum were achievable. The pelvic OARs were also delineated, using the RTOG guidelines, and included the rectum, large bowel, small bowel, bladder, penile bulb, and the femoral heads. The planning target volume (PTV) high was derived by a 5-mm expansion around the CTV high, except for a 4-mm expansion in the posterior direction. The PTV low was obtained by a 5-mm expansion around CTV low.

### Treatment Planning

Two sets of plans (IMPT and VMAT) were prepared for each patient with predefined dose-volume histogram (DVH) objectives for target volumes and OAR. Quantitative evaluation of plans was performed by means of a standardized DVH. For CTVs and PTVs, D99% and D2% (dose received by ≥ 99% and ≥ 2% of the volume, respectively) were reported as metrics for minimum and maximum doses. To complement the appraisal of D99% and D2%, V100% and V107% (the volume receiving ≥ 100%, and ≤ 107% of the prescribed dose, respectively) were reported. For OARs, the analysis included the mean dose, the maximum dose expressed as D2cc (Gy), and a set of appropriate volume metrics.

### Intensity-Modulated Proton Therapy

An optimized proton plan was generated by the Eclipse treatment-planning system. Spot spacing was set to 3 mm. The planning process involved the inverse optimization of dose distribution generated by a number of pencil beam spots scanning for cloud-covering targets and OARs and the modulating each beam spot with simultaneous tuning of spot energy and weight. Two opposed lateral beams were used in all cases. Plan quality and acceptability were assessed based on DVH parameters of target volumes and OARs.

### Volumetric-Modulated Arc Therapy

IMRT at our institution is delivered via VMAT. Moreover, VMAT has been widely used to treat prostate cancer, given its demonstrated plan quality and efficiency. An optimized VMAT plan was generated by the Eclipse treatment-planning system. The VMAT plans consisted of ≥ 2 coplanar arcs.

### Daily Proton Therapy

Treatment setup for daily proton beam therapy was identical to the simulation setup, including bladder and bowel preparation. Image-guided localization of the prostate was achieved with onboard orthogonal-kilovolt imaging of the intraprostatic carbon markers before treatment of each field.

When proton therapy was delivered with a daily setup based on the prostate position (using on-line matching of the intraprostatic carbon markers), there was uncertainty about the dose coverage of the regional pelvic nodes, which can be independent of prostate motion. To assess the adequacy of the CTV coverage during the 5-week course of proton therapy, a verification CT scan was acquired weekly, starting 1 day before the start of proton therapy. The CTV high and CTV low were propagated from the planning CT scan to the verification CT scans by matching intraprostatic carbon markers and pelvic bones, respectively. Coverage of CTVs was then evaluated on the weekly verification CT scans.

### Statistical Analysis

Various dosimetric parameters were compared between IMPT and VMAT plans. Percentage of the volume of the rectum, large bowel, small bowel, bladder, and femoral heads receiving between 10 and 70 Gy were evaluated and compared. The paired 2-sided Wilcoxon signed-rank test was used to compare IMPT versus VMAT plans. The level of statistical significance adopted was *P* < .05. All statistical analyses were performed with the statistical software R (R Foundation for Statistical Computing, Vienna, Austria).

## Results

### CTV and PTV Coverage

**Figure 1.**
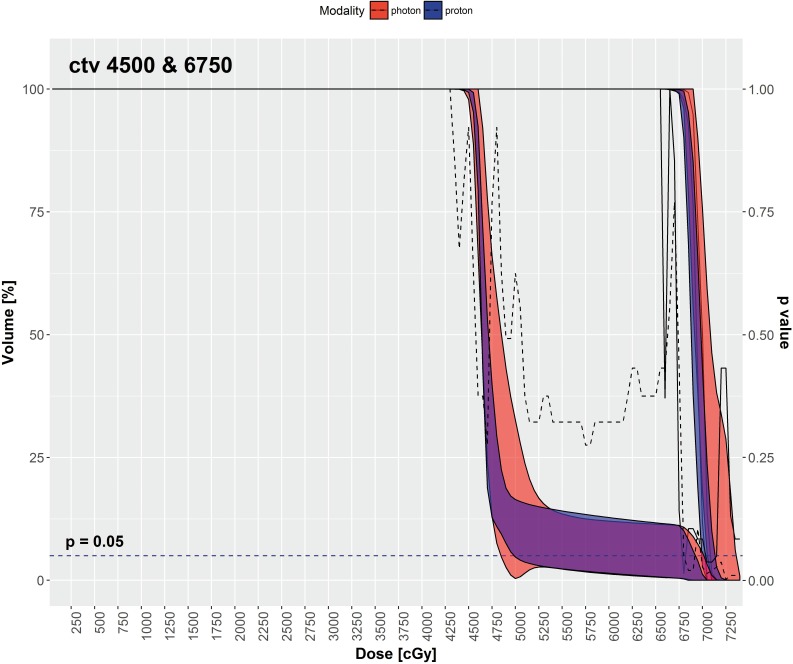
Target coverage—median DVH metrics with a 95% confidence interval boundary for IMPT (blue) versus VMAT (red). The solid (CVT high) and dashed lines (CTV low) are P values on a Wilcoxon signed-rank test, when DVHs were compared between IMPT and VMAT at each particular dose point. The right vertical axis represents P values. The blue-dashed, horizontal line denotes a P = 0.05. When the dashed and solid lines are below this horizontal line, it indicates that there is a statistically significant DVH difference between IMPT versus VMAT, with P < 0.05 at that particular dose point. Abbreviations: CVT, clinical target volume; DVH, dose-volume histogram; IMPT, intensity-modulated proton therapy; VMAT, volumetric-modulated arc therapy.

**Table 1** shows the mean values with standard deviations for the mean (cGy) dose, D99% (%), D95% (%), D2% (%), V95% (%), V100% (%), and V107% (%) of CTV high and CTV low for IMPT versus VMAT. The coverage of CTVs was adequate for both plans with > 99% of CTVs receiving the prescription doses. The mean values with standard deviations for the same DVH metrics of PTV high and PTV low are shown in **Table 2**. In both **Tables 1** and **2**, an asterisk is used to denote a statistically significant difference between IMPT and VMAT values on a Wilcoxon signed-rank test (*P* < .05). **Figure 1** depicts the median DVH values of the CTV high and CTV low at various dose points for each modality.

**Table 1.** Mean values with standard deviations for various DVH metrics of CTV high and CTV low for IMPT versus VMAT.

**Parameter**	**CTV high^a^**		**CTV low**	
	**IMPT**	**VMAT**	**IMPT**	**VMAT**
Mean (cGy)	6934.2 ± 67*	7013 ± 130.3*	4849.8 ± 159.7	4896.5 ± 227.1
V107% (%)	0.1 ± 0.2	10.5 ± 30	18.1 ± 11.5	29.9 ± 32.6
V100% (%)	99.4 ± 0.6	99.7 ± 0.6	99.5 ± 0.8	99 ± 2.7
V95% (%)	100 ± 0	100 ± 0	100 ± 0	100 ± 0
D99% (%)	100.6 ± 0.7	101.7 ± 2	101.7 ± 0.4	103.1 ± 1.7
D95% (%)	101.2 ± 0.8*	102.5 ± 1.8*	102.7 ± 0.3	104.1 ± 1.8
D2% (%)	104.5 ± 1.3	105.4 ± 2.1	137.4 ± 12.1	136.7 ± 12.9

**Abbreviations:** DVH, dose-volume histogram; CVT, clinical target volume; IMPT, intensity-modulated proton therapy; VMAT, volumetric-modulated arc therapy; V, volume receiving ≥ %; D, dose received by ≥ %.

a An asterisk (*) denotes a statistically significant difference between IMPT and VMAT values on a Wilcoxon signed-rank test (*P* < 0.05).

**Table 2.** Mean values with standard deviations for various DVH metrics of PTV high and PTV low for IMPT versus VMAT.

**Parameter**	**PTV high^a^**		**PTV low**	
	**IMPT**	**VMAT**	**IMPT**	**VMAT**
Mean (cGy)	6908.5 ± 50.3*	6979.4 ± 115.5*	4845.1 ± 160.8	4889.8 ± 215.8
V107% (%)	0.1 ± 0.2	8.6 ± 24.6	0 ± 0	0 ± 0.1
V100% (%)	93.8 ± 3.5	94.9 ± 2.4	5.9 ± 7.1	5.7 ± 6.9
V95% (%)	99.7 ± 0.5	99.6 ± 0.8	6.9 ± 7.6	6.6 ± 7.3
D99% (%)	97.5 ± 1.6	97.4 ± 2.2	98.8 ± 1.4	100.4 ± 1.9
D95% (%)	99.7 ± 0.9	100 ± 1.4	101.4 ± 0.5	102.9 ± 1.6
D2% (%)	104.6 ± 1.1	105.5 ± 2	140.9 ± 11.7	140.2 ± 12.2

**Abbreviations:** DVH, dose-volume histogram; PTV, planning target volume; IMPT, intensity-modulated proton therapy; VMAT, volumetric-modulated arc therapy; V, volume receiving ≥ %; D, dose received by ≥ %.

a An asterisk (*) denotes a statistically significant difference between IMPT and VMAT values on a Wilcoxon signed-rank test (*P* < 0.05).

### OARs: Rectum, Large bowel, Small bowel, Bladder, and Femoral heads

**Figure 2.**
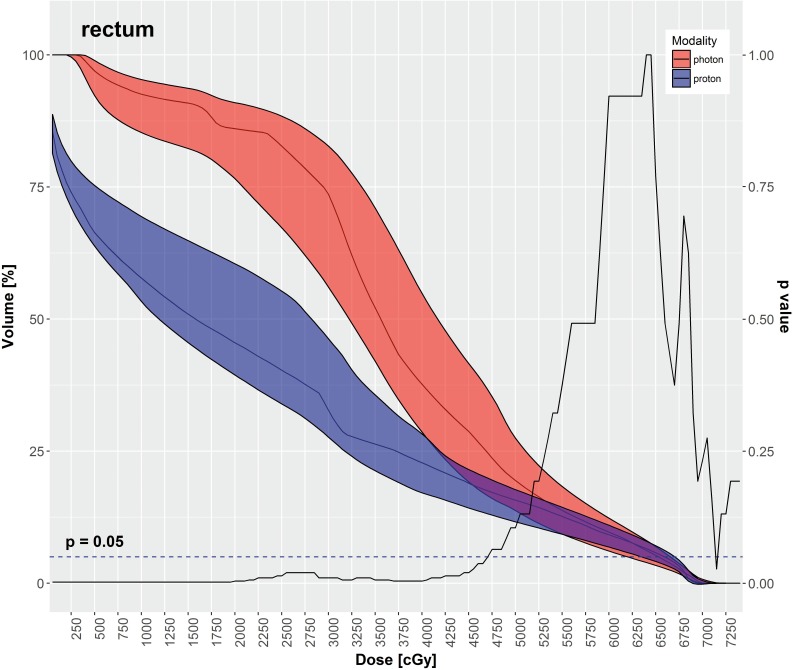
Rectum—median DVH metrics with a 95% confidence interval boundary for IMPT (blue) versus VMAT (red). The solid line is a P value on a Wilcoxon signed-rank test, when DVHs are compared between IMPT and VMAT at each particular dose point. The volume of rectum receiving ≤ 47.5 Gy was significantly less with IMPT. Abbreviations: DVH, dose-volume histogram; IMPT, intensity-modulated proton therapy; VMAT, volumetric-modulated arc therapy.

IMPT had significantly lower mean doses for most OARs in comparison to VMAT, as depicted in **Table 3**, including the rectum, large bowel, small bowel, and bladder. The mean dose to the femoral heads was significantly higher with IMPT in comparison to VMAT. **Table 3** also displays various DVH metrics with statistical comparison of IMPT versus VMAT. **Figures 2**[Fig i2331-5180-5-3-11-f03][Fig i2331-5180-5-3-11-f04][Fig i2331-5180-5-3-11-f05] through **6** graphically depict DVHs of the rectum, large bowel, small bowel, bladder, and femoral heads, respectively, at various dose points for each modality.

**Table 3.** Mean values for various DVH metrics of OARs for IMPT versus VMAT.

**Parameter**	**Bladder^a^**		**Rectum**		**Large bowel**		**Small bowel**		**Femoral heads**	
	**IMPT**	**VMAT**	**IMPT**	**VMAT**	**IMPT**	**VMAT**	**IMPT**	**VMAT**	**IMPT**	**VMAT**
Mean (cGy)	2852*	3726*	2347*	3668*	2155*	3014*	917*	2010*	1821*	1475*
V66 Gy (%)	3.6	4.0	4.8	4.3	0.0	0.0	0.0	0.0	0.0	0.0
V61 Gy (%)	6.3	6.9	8.3	8.4	0.2	0.0	0.0	0.0	0.0	0.0
V57 Gy (%)	8.1	9.2	10.7	11.8	0.3	0.0	0.0	0.0	0.0	0.0
V45 Gy (%)	16.6	24.0	17.9*	30.4*	5.1	11.7	3.7	4.3	0.0	0.0
V40 Gy (%)	23.7	42.4	22.7*	41.8*	19.3	32.5	8.0	11.1	0.3	0.1
V30 Gy (%)	40.2*	69.9*	37.0*	69.4*	44.4	52.3	14.1*	23.4*	13.5*	6.2*
V20 Gy (%)	70.3	86.6	49.9*	83.8*	55.8*	74.5*	21.3*	45.4*	61.6*	29.7*
V10 Gy (%)	84.8*	100.0*	61.4*	90.3*	62.1*	92.3*	28.3*	71.2*	68.8	67.6

**Abbreviations:** DVH, dose-volume histogram; OARs, organs at risk; IMPT, intensity-modulated proton therapy; VMAT, volumetric-modulated arc therapy; cGy, centigray; V, volume receiving ≥ %.

a An asterisk (*) denotes a statistically significant difference between IMPT and VMAT values on a Wilcoxon signed-rank test (*P* < 0.05).

**Figure 3. i2331-5180-5-3-11-f03:**
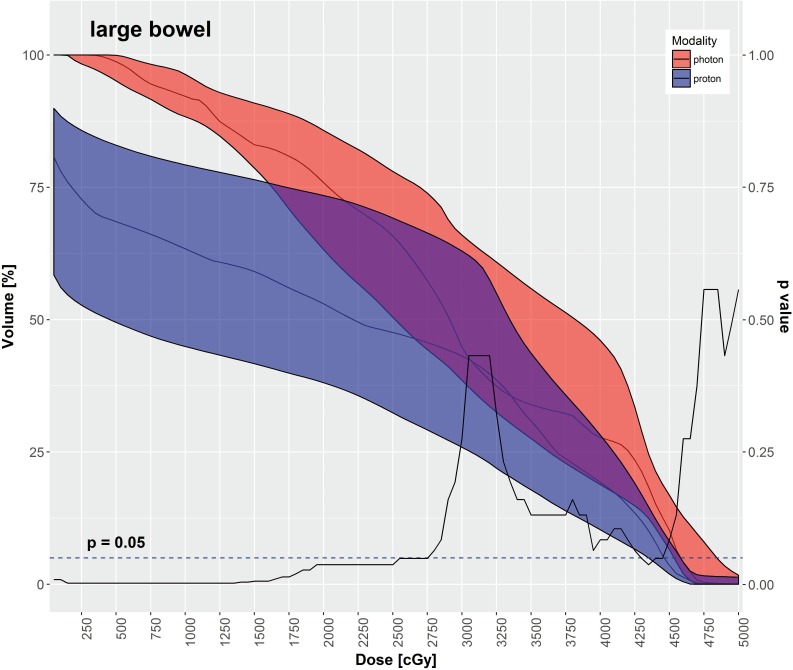
Large bowel—median DVH metrics with a 95% confidence interval boundary for IMPT (blue) versus VMAT (red). The solid line is a P value on a Wilcoxon signed-rank test, when DVHs are compared between IMPT and VMAT at each particular dose point. The volume of large bowel receiving ≤ 27.5 Gy was significantly less with IMPT. Abbreviations: DVH, dose-volume histogram; IMPT, intensity-modulated proton therapy; VMAT, volumetric-modulated arc therapy.

**Figure 4. i2331-5180-5-3-11-f04:**
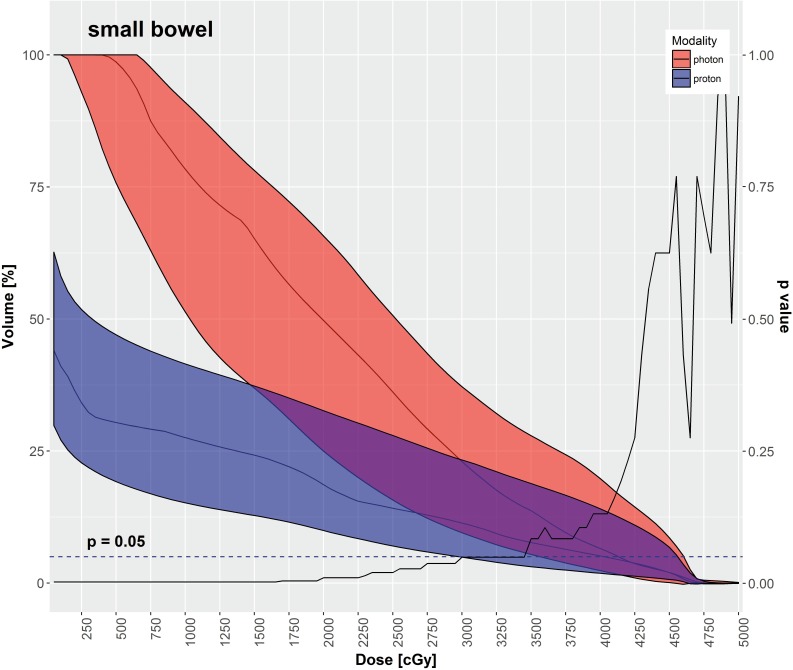
Small bowel—median DVH metrics with a 95% confidence interval boundary for IMPT (blue) versus VMAT (red). The solid line is a P value on a Wilcoxon signed-rank test, when DVHs are compared between IMPT and VMAT at each particular dose point. The volume of small bowel receiving ≤ 30 Gy was significantly less with IMPT. Abbreviations: DVH, dose-volume histogram; IMPT, intensity-modulated proton therapy; VMAT, volumetric-modulated arc therapy.

**Figure 5. i2331-5180-5-3-11-f05:**
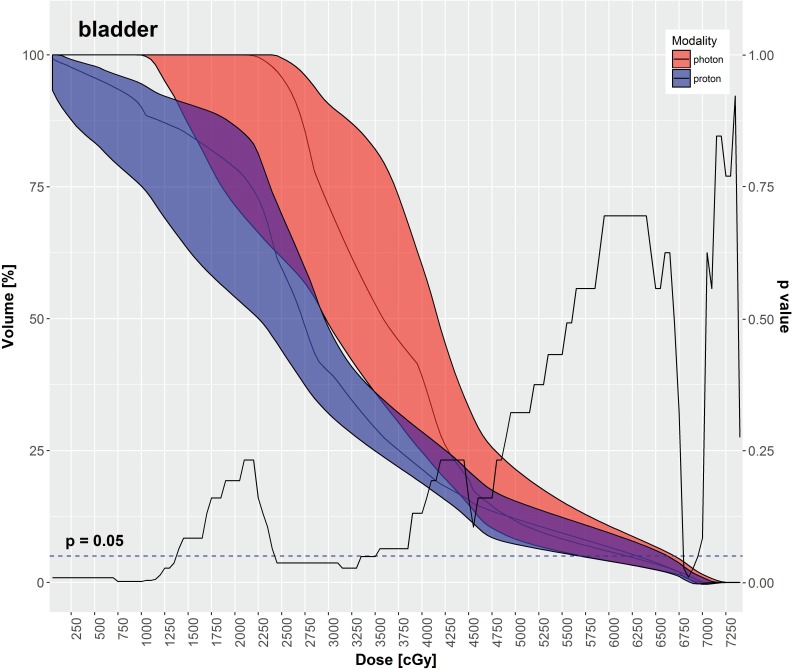
Bladder—median DVH metrics with a 95% confidence interval boundary for IMPT (blue) versus VMAT (red). The solid line is a P value on a Wilcoxon signed-rank test, when DVHs are compared between IMPT and VMAT at each particular dose point. The volume of bladder receiving between 25 Gy and 37.5 Gy, and ≤ 13.75 Gy was significantly less with IMPT. Abbreviations: DVH, dose-volume histogram; IMPT, intensity-modulated proton therapy; VMAT, volumetric-modulated arc therapy.

**Figure 6.**
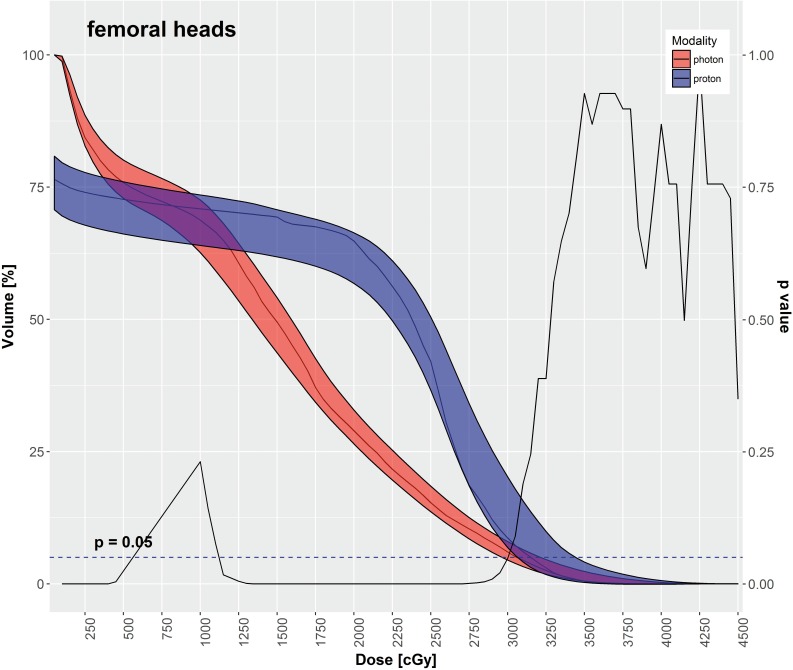
Femoral heads–median DVH metrics with a 95% confidence interval boundary for IMPT (blue) versus VMAT (red). The solid line is a P value on a Wilcoxon signed-rank test, when DVHs are compared between IMPT and VMAT at each particular dose point. The volume of femoral heads receiving between 30 Gy and 11.25 Gy was significantly less with VMAT, whereas that receiving < 5.75 Gy was less with IMPT. Abbreviations: DVH, dose-volume histogram; IMPT, intensity-modulated proton therapy; VMAT, volumetric-modulated arc therapy.

As shown in **Table 3** and **Figures 2** through **6**, the DVH metrics favored IMPT over VMAT, except for the femoral heads. In general, IMPT plans did not produce the typical low-dose “bath” to the pelvis seen with VMAT plans. For the femoral heads, the mean dose and percentage of volumes receiving 11.25 to 30 Gy were greater with IMPT plans. Those higher DVH metrics were primarily a by-product of 2 opposed lateral beams used for a typical IMTP plan.

## Discussion

For high-risk prostate cancer, radiation treatment volumes often include elective pelvic lymph nodes because of an increased risk of occult lymph node metastasis. The efficacy of pelvic lymph node coverage for unfavorable, intermediate or high-risk prostate cancer remains an open question, currently being investigated by RTOG 0924 (NCT01368588). Recent evidence in the setting of salvage RT showed a significant benefit for biochemical free survival with the addition of pelvic nodal radiation [9]. However, treatment of the pelvic lymph nodes may come at the cost of increased toxicity. The additive toxicity of elective pelvic coverage in the primary setting with IMRT was recently evaluated in the PIVOTAL trial. The increase in toxicity with elective nodal coverage compared with prostate-only coverage was mild to moderate. Patients receiving elective nodal coverage had an approximately 20% greater rate of acute, grade 2 gastrointestinal toxicity [10].

There have been no prospective studies reported to date, to our knowledge, evaluating the efficacy and toxicity of proton beam therapy in the setting of unfavorable, intermediate or high-risk prostate cancer in which target volumes are expanded to cover the regional pelvic lymph nodes. Recently, Chuong et al [11] reported the acute toxicity of proton beam therapy on a subset of patients with prostate cancer who had the regional pelvic lymph nodes and the prostate and/or seminal vesicles irradiated, from a multi-institutional, prospective database. They reported low acute toxicity rates with 2.4% grade 2 gastrointestinal toxicity and no grade 3 toxicity. Currently, our group is conducting a prospective study for patients with unfavorable, intermediate or high-risk prostate cancer in which both the regional pelvic lymph nodes and the prostate and/or seminal vesicles are treated with IMPT with a pencil-beam scanning technique. As part of the evaluation of this application of proton beam therapy, we conducted a comparative dosimetric study comparing IMPT and VMAT to assess the extent of dosimetric advantage or disadvantage of IMPT.

IMPT spared more OARs in the pelvis than the VMAT did in our study. The mean doses to the rectum, large bowel, small bowel, and bladder were significantly decreased with IMPT as compared with VMAT. Additionally, the volumes of those OARs receiving low-to-medium radiation doses were significantly smaller with IMPT plans compared with VMAT plans. That difference is readily evident in the shapes of the DVH curves for each OAR, shown in **Figures 2** through **6**. Although VMAT can deliver highly conformal, high-dose target coverage, this is achieved by spreading out the low-to-medium dose over a larger volume of nontargeted tissues. In contrast, IMPT is able to reduce the dose to nontargeted tissues significantly and deliver equally conformal, high-dose target coverage because of its distinct advantage of having minimal dose distal to the targets.

It is plausible that this large-dose reduction in the pelvic organs by IMPT can translate into lower gastrointestinal and genitourinary toxicity. In our cohort, the mean dose to the rectum was decreased by 36%, and the percentage volume receiving ≤ 45 Gy was significantly reduced with IMPT. In the dose-escalation study by the MD Anderson Cancer Center, DVH metrics revealed a continuous dose effect for rectal toxicity, and differences in the lower-dose region were more predictive of rectal morbidity than the higher-dose region [12]. The small bowel is the dose-limiting organ with the lowest dose tolerance in the pelvis. Quantitative Analyses of Normal Tissue Effects in the Clinic guidelines recommend restricting the volume the small bowel receives to ≤ 15 Gy when individual bowel loops are used for contouring, and the volume receiving 45 Gy when the peritoneal cavity or a bowel bag is used for contouring [13]. However, this is often not achievable even with IMRT technique [11]. In our study, the mean dose to the small bowel was decreased by 54% with IMTP compared with VMAT. Similarly, our study shows that IMPT plans significantly reduced the volume of the large bowel and the bladder receiving low-to-medium radiation doses. This dosimetric advantage for the pelvic organs indicates that IMPT is a technical advance, which could improve the therapeutic ratio of elective pelvic lymph node coverage for patients with prostate cancer. Furthermore, these findings are consistent with prior analyses [14–17] showing a reduction in dose to OARs with proton therapy, including previous series investigating the coverage of pelvic lymph nodes.

The unresolved question is whether a large reduction in low-to-medium doses to the pelvic organs by IMPT actually reduces gastrointestinal and genitourinary toxicity, in comparison with IMRT. A prior comparative effectiveness study [18] found no differences in overall quality of life but noted decreased rectal urgency and frequent bowel movements with proton therapy as compared with IMRT, though patients receiving elective nodal coverage were excluded from this analysis. A phase III study is ultimately required to address this important question. Our current, single-arm study of IMPT is designed to evaluate the acute and late side effects of IMPT, using both patient-reported and physician-reported Common Terminology Criteria for Adverse Events. Its outcomes can be the basis for launching a multi-institutional, comparative phase III study specifically addressing whole-pelvic radiation therapy. The Patient Centered Outcomes Research Institute is currently investigating proton versus photon therapy, with a primary endpoint of the expanded prostate cancer index composite domain score, 2 years after the completion of RT (NCT03561220). This trial will generate prospective data for the subset of patients receiving whole-pelvic radiation therapy.

Of note, in our study, the mean dose and the percentage volume of the femoral heads receiving 20 to 30 Gy were higher with IMPT plans. In addition, maximum point doses were generally greater for IMPT compared with VMAT. These higher DVH metrics were primarily a byproduct of 2 opposed lateral beams used for a typical IMTP plan. This disparate effect of IMPT can be eliminated by adding another beam in a different direction (such as a posterior oblique beam). Furthermore, the clinical effect of the increased dose to the femoral heads in the IMTP plans may not be significant because the mean dose to the femoral heads (1821 cGy) was well below the 45 Gy generally considered a clinically acceptable dose.

A limitation of our analysis is that all photon plans were generated specifically for this dosimetric study. However, the quality of all photon plans was reviewed by radiation oncologists specializing in the treatment of genitourinary malignancy, met departmental criteria, and all were planned by dosimetrists using standardized departmental methodology. Additionally, the dosimetric differences observed here with IMPT are in the setting of 2 opposed lateral fields for each IMPT plan. More sophisticated IMPT approaches, including the use of additional anterior or posterior oblique beam(s) could result in even greater differences in this cohort between IMPT and VMAT plans. Nevertheless, even with opposed lateral fields alone, as these patients were treated, significant differences in dose to OARs were noted.

In summary, the dosimetric data presented here provide achievable dose constraints for whole-pelvic radiation therapy with IMPT as a reference for future studies, and the comparative results provide further rational for the prospective evaluation of IMPT versus IMRT, especially in the setting of elective pelvic lymph node coverage.

## Conclusion

Intensity-modulated proton therapy can significantly reduce low-to-medium doses to the rectum, large bowel, small bowel, and bladder, in comparison with VMAT. This dosimetric advantage of IMPT may translate into a decreased risk of gastrointestinal and genitourinary toxicity for patients receiving pelvic lymph node irradiation. Further study is warranted to correlate a dosimetric advantage of IMPT with clinical outcomes.
